# Impact of chemotherapy and radiotherapy on the survival of elderly esophageal cancer patients undergoing surgery: a SEER database analysis

**DOI:** 10.1186/s12876-021-02016-9

**Published:** 2021-11-18

**Authors:** Xinrong Li, Jin Zhang, Chenxiao Ye, Junquan Zhu, Kaibo Guo, Yong Guo

**Affiliations:** 1 Department of Integrative Medicine and Medical Oncology, Shengzhou People’s Hospital (the First Affiliated Hospital of Zhejiang University Shengzhou Branch), Shengzhou, 312400 Zhejiang People’s Republic of China; 2 The First Clinical College, Zhejiang Chinese Medical University, Hangzhou, 310053 Zhejiang People’s Republic of China; 3 Department of Medical Oncology, The First Affiliated Hospital of Zhejiang Chinese Medical University, Hangzhou, 310006 Zhejiang People’s Republic of China

**Keywords:** Elderly patient, Esophageal cancer, Nomogram, Predictive model, Cancer-specific death

## Abstract

**Background:**

Esophageal cancer (EC) is a common and lethal carcinoma; however, the effectiveness and feasibility of the chemo- and radio-therapy (CRT) for the elderly patients (≥ 70 years) with surgery have not been fully discussed. The purpose of this study was to investigate the potential effect of CRT on the prognosis.

**Methods:**

A total of 1085 patients (534 CRT patients vs. 551 non-CRT patients) from 1998 to 2016 were collected from the Surveillance, Epidemiology, and End Results database according to the inclusion and exclusion criteria. Using the competing risk regression and survival analysis, an overall estimation of the effectiveness of CRT was performed on a well-balanced cohort via performing propensity score matching. Then, the specific impact of CRT on high- (n = 557) and low-risk (n = 528) cohorts derived from the nomogram’s risk quantification for every patient were further evaluated respectively. Additionally, the advantages of the nomogram model and the conventional tumor, node, metastasis (TNM, 6th revision) staging system were compared.

**Results:**

A better survival outcome was observed among patients receiving both surgery and CRT than those who underwent surgery alone (HR: 0.55, 95% CI 0.45–0.68, *P* < 0.001), especially for those with tumors characterized by poor differentiation, large tumor size, advanced T staging, lymphatic metastasis, and distant metastasis (HR: 0.48, 95% CI 0.39–0.59, *P* < 0.001), while no benefit was observed among the low-risk patients. Furthermore, the newly established nomogram model might be better than the TNM (6th revision) staging system but more data needed.

**Conclusion:**

Aggressive treatments, such as surgery, chemotherapy, and radiotherapy, were considered effective for selected elderly patients with EC according to the newly established nomogram model.

**Supplementary Information:**

The online version contains supplementary material available at 10.1186/s12876-021-02016-9.

## Background

Esophageal cancer (EC) is a common upper gastrointestinal tract carcinoma with high morbidity and mortality worldwide, with approximately 5.5% of new digestive tumor cases and 2.7% of tumor mortality in 2020 [1]. The incidence is considered to be increasing [2], especially in some developing and deprived regions such as East Asia and Southern and Eastern Africa [3]. In most parts of North America and Europe, the incidence of esophageal squamous cell carcinoma is decreasing, while the incidence of esophageal adenocarcinoma is increasing [4, 5]. For example, in the United States, the age-adjusted incidence rate of esophageal adenocarcinoma increased from 1.8 per 100,000 in 1987–1991 to 2.5 per 100,000 in 1992–1996 [6]

Recently, to improve the quality of life and prolong the survival time, progress on treatments has been made, including minimally invasive surgery for early stage EC [7], neoadjuvant chemotherapy for potentially resectable EC histologically confirmed as squamous cell carcinoma [8] or adenocarcinoma [9] at a locally advanced stage, and immune checkpoint inhibitors for advanced EC [10]. Furthermore, to increase the resection rate in the early stage (T stage > T1), to prolong survival, and to relieve the uncomfortable symptoms in the advanced stage, the combination of chemotherapy and radiotherapy is the most preferred treatment in addition to surgery according to the National Comprehensive Cancer Network guidelines, except for patients with poor physical conditions [11]. Despite this, owing to its extremely aggressive behaviors and insensitivity to the conventional treatment, the prognosis is still poor, accounting for 15–25% of the overall 5-year survival rate worldwide [2]. Numerous studies [12–14] have indicated that old age is a risk factor for reducing the survival probability of patients. Because EC patients aged ≥ 70 years are not considered candidates in regular clinical trials [15] due to their declining physiological function and underlying comorbidities, data concerning the management is limited and the principle of treatment remains unclear [16]. However, with the aging global population, it is urgent to explore the roles of conventional treatments, including chemotherapy and radiotherapy, in elderly patients with EC based on the existing data.

To facilitate clinical decision-making for elderly patients with EC, we analyzed the impact of chemo- and radio-therapy (CRT, not to be confused with chemoradiotherapy) on the elderly patients with EC undergoing surgery based on a cohort from the Surveillance Epidemiology and End Results (SEER) database. First, we estimate the general impact of CRT based on two well-comparable cohorts processed by propensity score matching (PSM). To further identify the specific patient subgroup that might benefit from CRT, a nomogram model was established to measure the risk for every patient based on individual characteristics, which could be further labeled as high risk (high score) or low risk (low score) according to the median score of the whole cohort. Finally, predictive models were created to measure the effects of CRT on a specific population.

## Methods

The study was approved by the Institutional Ethical Committee of Shengzhou People's Hospital (approval number: 2020-03).

### Study population

**Fig. 1:**
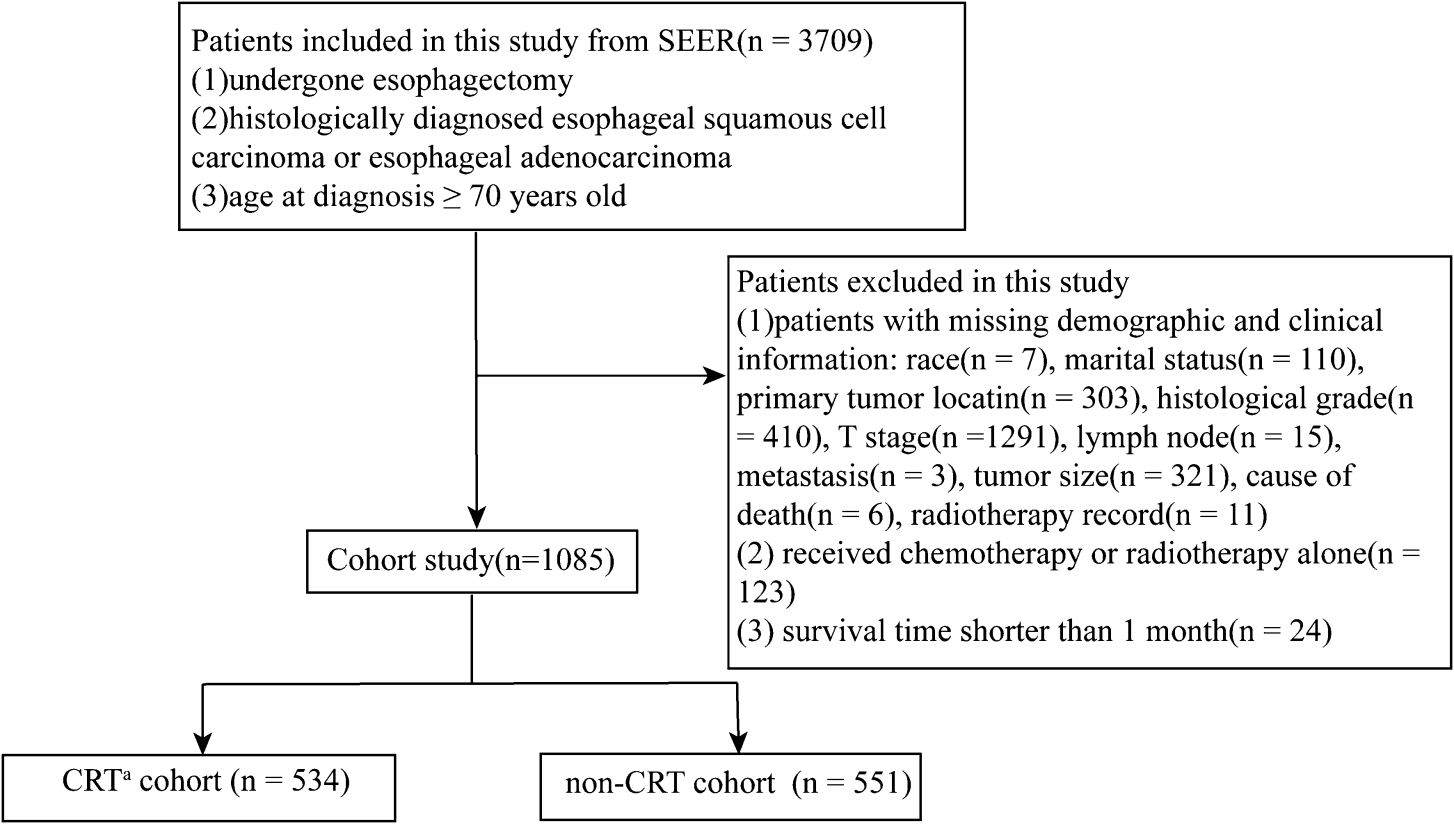
The flowchart of the present study. ^a^*CRT* chemo- and radio-therapy

This study collected data from the patients registered in the United States SEER cancer registries from 1998 to 2016. The inclusion criteria were as follows: (a) patients who underwent esophagectomy; (b) histologically diagnosed esophageal squamous cell carcinoma or esophageal adenocarcinoma; and (c) age at diagnosis ≥ 70 years. The exclusion criteria were as follows: (a) patients with missing demographic or clinical information (sex, race, histological type, tumor size, etc.); (b) received chemotherapy or radiotherapy alone; and (c) survival time shorter than 1 month. Following the inclusion and exclusion criteria, 1085 eligible patients (534 CRT patients vs. 551 non-CRT patients) were selected for the further analysis (Fig. 1). The median age of the patients was 75 years (rang 72–78 years).

### Data collection

Cases histologically confirmed with EC according to the International Classification of Diseases in Oncology (ICD‐O‐3) from 1998 to 2016 were identified from the SEER database, an open database available at https://seer.cancer.gov/, using SEER*Stat Software (version 8.3.8) [17]. The “SEER Combined Stage Group (2016+)” was derived from the patients from 2004 to 2015, who were also classified by the tumor, node, metastasis (TNM, 6th revision) staging system, indicating that the above two staging standards could be combined to evaluate the disease. The tumor size was exactly determined by the record including “EOD 10—size (1988–2003),” “CS tumor size (2004–2015),” or “tumor Size Summary (2016+),”, because the measurement standards in the past 30 years were different.

### Covariates

To facilitate statistical analysis, some demographics and clinical covariates were modified based on our clinical and research experiences: race was defined as “white” or “other races (black, American Indian/Alaska Native, Asian/Pacific Islander),” histological type was defined as “squamous cell carcinoma” or “adenocarcinoma,” and histological grade was defined as “I + II (well or moderate differentiation grade)” or “III + IV (poorly or undifferentiation grade) grade.” Then, a matrix was built containing the information for race, age at diagnosis, marital status at diagnosis, primary tumor location, histological type and grade, TNM staging system (6th), tumor size, treatment record, survival status, survival time, and cause of death for every patient.

### Statistical analysis

R-software packages of “MatchIt” and “cobalt” were used to conduct the PSM; packages of “survival,” “cmprsk,” and “survminer” were used to establish competing risk models; packages of “mstate,” “riskRegression,” and “rmda” were used to build nomogram, calibration curves, and decision curve analysis. Categorical variables described as counts and relative percentages were compared using the χ^2^ test, and *P* < 0.05 was considered statistically significant.

Next, the whole-population cohort was divided into CRT and non-CRT groups through 1:1 nearest neighbor PSM with a caliper of 0.08. The sub-distribution hazard ratio (sHR) and hazard ratio (HR) were calculated to estimate the probability of cancer-specific death (CSD) and overall survival (OS), and the results were presented using the cumulative incidence function curves and Kaplan–Meier curves [18, 19], respectively. Love-plot [20] and χ^2^ tests were performed to assess the effectiveness of PSM. Next, the whole-population cohort was randomized into training and validation sets at a ratio of 7:3. Based on the training set, univariate and multivariate competing risk models were established to select the key risk factors for CSD. Then, high- and low-risk groups were derived according to the risk score from the nomogram model built on the above risk factors. The impact of CRT on the two specific groups was then estimated. Furthermore, in both the training and validation sets, concordance indexes (c-index) were calculated to show the discrimination, and calibration curves were drawn to measure the consistency between the actual and expected values. Finally, a decision curve analysis was conducted to measure the superiority of the nomogram model over the conventional TNM staging system.

## Results

### Analysis in the post-match cohort

Based on the inclusion and exclusion criteria, 1085 patients were selected and grouped into the CRT and non-CRT cohorts. Propensity matching based on the individual characteristics of patients was then performed to the two cohorts; some patients that were not matched were censored. Eventually, 278 patients in the CRT cohort and 278 patients in the non-CRT cohort were included for further analysis. Before matching, only the distributions of sex (*P* = 0.2), race (*P* = 0.372), primary tumor location (*P* = 0.225), tumor histological type (*P* = 0.154), and distant metastasis (*P* = 0.173) between the CRT and non-CRT cohorts were similar. However, all variables’ distributions became balanced after matching (Table 1, Additional file 1: Fig. S1), indicating a good comparability between the two post-match cohorts.

**Table 1:** Clinical characteristics before and after PSM according to CRT or not

Variables	Before matching		*P*^a^ value	After matching		*P* value
	CRT N = 534 (%)	Non-CRT N = 551 (%)		CRT N = 277 (%)	Non-CRT N = 277 (%)	
Sex			0.2			0.84
Male	443 (82.96%)	426 (77.31%)		212 (76.53%)	214 (77.26%)	
Female	91 (17.04%)	125 (22.69%)		65 (23.47%)	63 (22.74%)	
Race			0.372			0.659
White	498 (93.26%)	506 (91.83%)		250 (90.25%)	253 (91.34%)	
Other races^b^	36 (6.74%)	45 (8.17%)		27 (9.75%)	24 (8.66%)	
Marriage			0.004			0.154
Married	386 (72.28%)	354 (64.25%)		188 (67.87%)	172 (62.09%)	
Unmarried	148 (27.72%)	197 (35.75%)		89 (32.13%)	105 (37.91%)	
Location			0.225			0.31
Upper third	10 (1.87%)	15 (2.72%)		8 (2.89%)	10 (3.61%)	
Middle third	82 (15.36%)	102 (18.51%)		62 (22.38%)	48 (17.33%)	
Low third	442 (82.77%)	434 (78.77%)		207 (74.73%)	219 (79.06%)	
Histology			0.154			0.089
SCC	106 (19.85%)	129 (23.41%)		87 (31.41%)	69 (24.91%)	
Adenocarcinoma	428 (80.15%)	422 (76.59%)		190 (68.59%)	208 (75.09%)	
Grade^c^			0.009			0.173
I + II	278 (52.06%)	330 (59.89%)		153 (55.23%)	137 (49.46%)	
III + IV	256 (47.94%)	221 (40.11%)		124 (44.77%)	140 (50.54%)	
Size			0			0.341
< 33 mm	354 (66.29%)	196 (35.57%)		171 (61.73%)	160 (57.76%)	
≥ 33 mm	180 (33.71%)	355 (64.43%)		106 (38.27%)	117 (42.24%)	
T stage			0			0.08
T1	59 (11.05%)	298 (54.08%)		57 (20.58%)	63 (22.74%)	
T2	91 (17.04%)	80 (14.52%)		64 (23.1%)	48 (17.33%)	
T3	347 (64.98%)	160 (29.04%)		133 (48.01%)	153 (55.23%)	
T4	37 (6.93%)	13 (2.36%)		23 (8.3%)	13 (4.69%)	
Lymph node			0			0.932
Positive	371 (69.48%)	404 (73.32%)		138 (49.82%)	139 (50.18%)	
Negative	163 (30.52%)	147 (26.68%)		139 (50.18%)	138 (49.82%)	
Metastasis			0.173			0.6
Yes	25 (4.68%)	17 (3.09%)		19 (6.86%)	16 (5.78%)	
No	509 (95.32%)	534 (96.91%)		258 (93.14%)	261 (94.22%)	

*CRT* chemo- and radio-therapy, *SCC* squamous cell carcinoma

^a^The *P* values of comparing CRT and non-CRT calculated by the using χ^2^ test

^b^Other races: Black, American Indian/Alaska Native, Asian/Pacific Islander

^c^I: well differentiation; II: moderate differentiation; III: poor differentiation; IV: undifferentiation

**Fig. 2:**
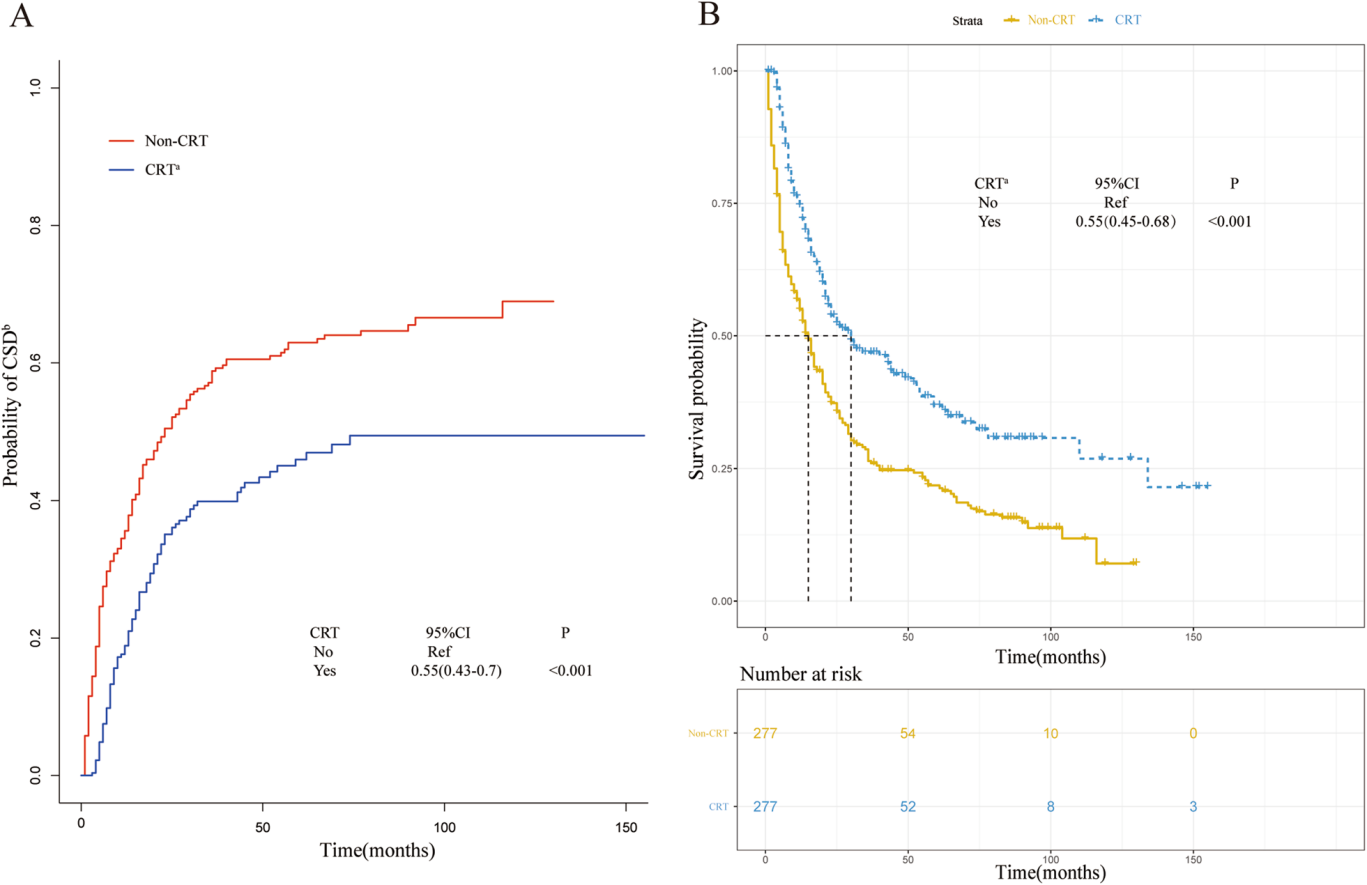
**a** Cumulative incidence estimates of cancer-specific death for patients with or without chemo- and radio-therapy (CRT) in the post-match cohort; **b** Overall survival of the post-match population with or without CRT. ^a^*CRT* chemo- and radio-therapy, ^b^*CSD* cancer-specific death

The competing regression and Cox regression models were constructed to identify the patients who received CRT that had lower probabilities of CSD (Fig. 2a, sHR: 0.55, 95% CI 0.43–0.7, *P* < 0.001) and better survival outcomes (Fig. 2b, CRT vs. non-CRT, HR: 0.55, 95% CI 0.45–0.68, *P* < 0.001) than those who did not receive CRT.

### Analysis in the pre-match cohort

To further study the effect of CRT on specific elderly (≥ 70 years) patients, the 1085 patients were randomly divided into training (n = 761) and validation (n = 324) sets. In the training set, the univariate and multivariate analysis of competing risk models were performed to estimate probabilities of CSD; as a result, poorly or un-differentiated grade, tumor size ≥ 33 mm, T stage > T1, positive lymph nodes, and metastasis diseases had been associated with increased risk of CSD (Table 2). In addition to the key factors mentioned above, the primary tumor location, which is considered a vital factor for prognosis [21], was also selected for further analysis.

**Table 2:** Sub-distribution hazard ratio (sHR) of characteristics for cancer-specific death in univariate and multivariate competing risk models

Characteristics	Univariate analysis			Multivariate analysis		
	sHR	95% CI	*P* value	sHR	95% CI	*P* value
Race						
White	1 (Ref)					
Other races^a^	1.21	0.85–1.72	0.29			
Sex						
Male	1 (Ref)					
Female	1.09	0.85–1.4	0.5			
Marriage						
Unmarried	1 (Ref)					
Married	1.06	0.83–1.34	0.65			
Location						
Upper third	1 (Ref)					
Middle third	1.1	0.55–2.18	0.8			
Low third	0.82	0.42–1.58	0.55			
Histology						
Adenocarcinoma	1 (Ref)					
SCC	1.1	0.85–1.42	0.46			
Grade^b^						
I + II	1 (Ref)			1 (Ref)		
III + IV	1.93	1.56–2.4	< 0.001	1.57	1.26–1.96	< 0.001
Size						
< 33 mm	1 (Ref)			1 (Ref)		
≥ 33 mm	2.14	1.71–2.68	< 0.001	1.34	1.04–1.75	0.026
T stage						
T1	1 (Ref)			1 (Ref)		
T2	1.95	1.35–2.81	< 0.001	1.5	1.01–2.22	0.046
T3	3.12	2.33–4.18	< 0.001	1.95	1.36–2.81	< 0.001
T4	6.14	3.8–9.93	< 0.001	3.3	1.88–5.76	< 0.001
Lymph node						
Negative	1 (Ref)			1 (Ref)		
Positive	2.45	1.96–3.06	< 0.001	1.51	1.17–1.96	0.002
Metastasis						
No	1 (Ref)			1 (Ref)		
Yes	2.58	1.64–4.06	< 0.001	1.68	1.07–2.65	0.024

*CI* confidence interval, *SCC* squamous cell carcinoma

^a^Other races: Black, American Indian/Alaska Native, Asian/Pacific Islander

^b^I: well differentiation; II: moderate differentiation; III: poor differentiation; IV: undifferentiation

**Fig. 3:**
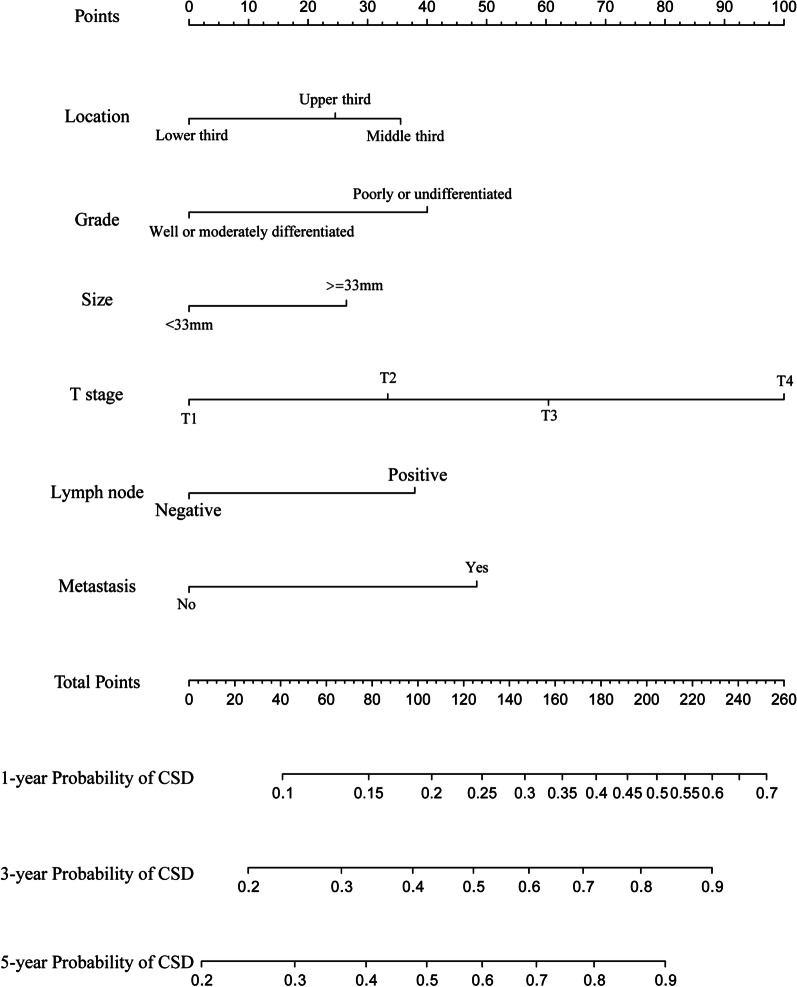
Nomogram predicting 1-, 3-, and 5-year probabilities of cancer-specific death for esophageal cancer patients based on the training cohort

**Fig. 4:**
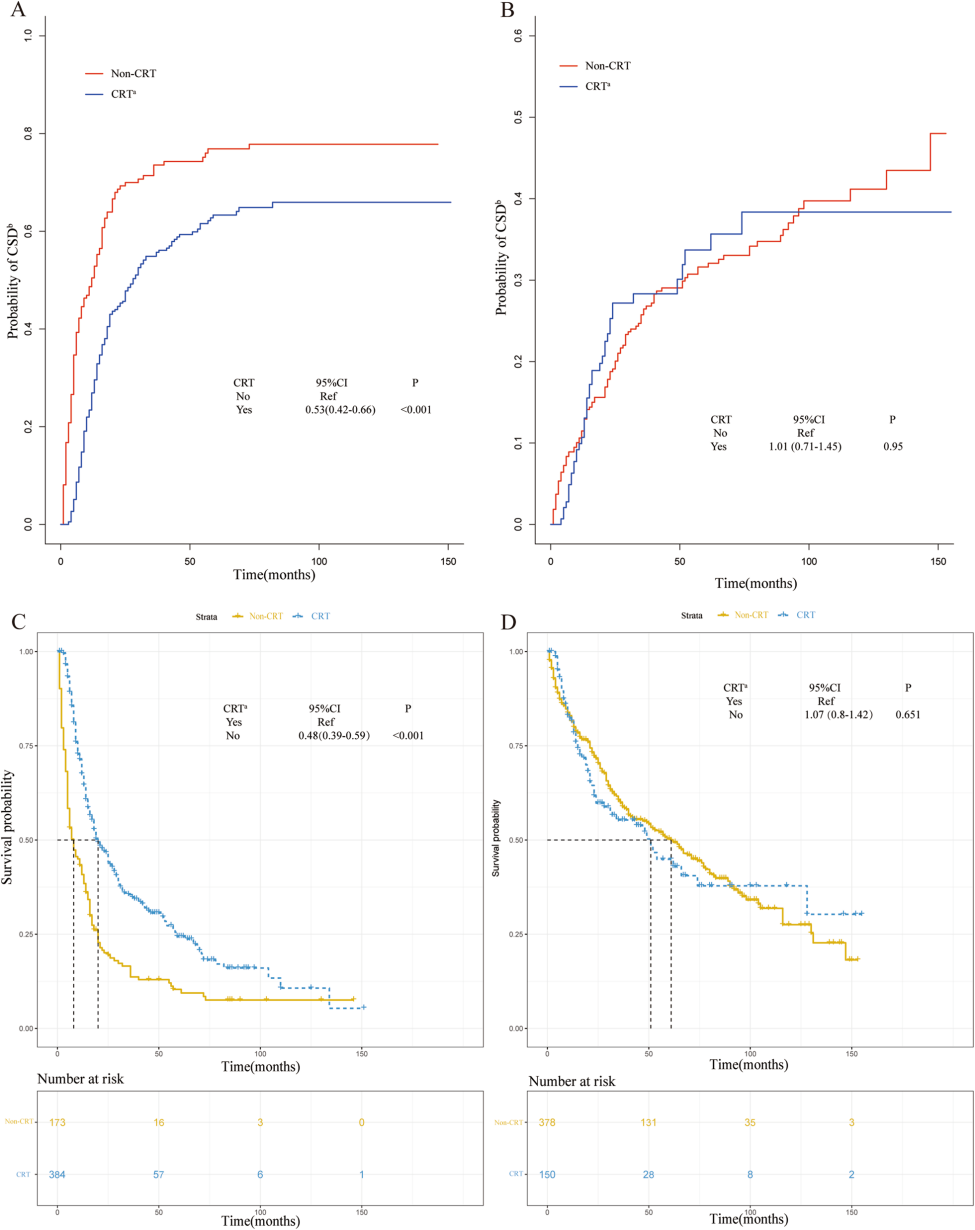
**a** Cumulative incidence estimates of cancer-specific death (CSD) for esophageal cancer (EC) patients with or without chemo- and radio-therapy (CRT) in the high-risk group. **b** Cumulative incidence estimates of CSD for EC patients with or without CRT in the low-risk group. **c** Overall survival (OS) of EC patients with or without CRT in the high-risk group; **d** OS of EC patients with or without CRT in the low-risk group. ^a^*CRT* chemo- and radio-therapy, ^b^*CSD* cancer-specific death

A nomogram was established based on the variables, including primary tumor location, histological grade, tumor size, T stage, lymphatic status, and distant metastasis, with the purpose of predicting the possibility of CSD in the next 1, 3, and 5 years (Fig. 3). Based on the risk score for each person derived from the nomogram, 1085 patients could be separated into high- (n = 557) and low-risk (n = 528) cohorts by a median score of 98 (high risk, score ≥ 98; low risk, score < 98). Next, the competing regression model was used to show that high-risk patients whose tumors were characterized as having poor differentiation, large tumor size, advanced T staging, lymphatic metastasis, and distant metastasis were more likely to benefit from CRT for lower probability of CSD (Fig. 4a, sHR: 0.53, 95% CI 0.42–0.66, *P* < 0.001) and better overall survival outcome (Fig. 4c, HR: 0.48, 95% CI 0.39–0.59, *P* < 0.001). However, no marked benefits were observed among the low-risk patients who received additional treatment (Fig. 4b, sHR: 1.01, 95% CI 0.71–1.45, *P* = 0.95; Fig. 4d, HR: 1.07, 95% CI 0.8–1.42, *P* = 0.651). In addition, the calibration curves showed that the 1-, 3-, and 5-year predictive probabilities of CSD matched well with the actual ones both in the training (Additional file 2: Fig. S2A, C, and E) and validation (Additional file 2: Fig. S2B, D, and F) sets. Furthermore, the decision curve analysis (Additional file 3: Fig. S3A–C) and the c-indexes for the competing risk regression model (0.636 and 0.648 in the training and validation sets, respectively) were just slightly higher than those for the TNM staging system (0.629 and 0.635 in the training and validation sets, respectively), indicating the discriminative superiority of the nomogram model over the TNM model for predicting survival outcomes needed more data to be proven.

## Discussion

With the extension of life expectancy, more EC patients would be present in those aged > 60 years [22]. A Chinese study indicated an increased incidence and mortality among the patients aged > 70 years compared to other age stratifications [23], leading to a sharp increase in healthcare costs in both developing [24] and developed [25] countries. However, guidelines that specifically address the management of elderly patients with EC are rare, and only a few studies refer to the topics of evaluation and treatment for the elderly.

In the present study, based on the post-PSM cohort, we found that CRT could have a positive impact on CSD and OS in elderly patients. Univariate and multivariate competing regression models were performed to identify poor histological differentiation, and advanced T stage and positive lymph node status were significantly correlated with higher probability of CSD. Furthermore, tumor size and distant metastasis were also considered key factors in predicting the prognosis of EC. Kamel et al. indicated that the size of malignancy (HR = 1.005) was a significant independent predictor of CSD in T1N0M0 patients based on the SEER database [26]. Malnutrition status was correlated with poor survival [27], while the esophageal tumor size was significantly correlated with nutritional status, as measured by the prognostic nutritional index (*P* = 0.016) [28]. Furthermore, larger tumor size was a predictive factor for identifying patients with EC who might have a higher rate of resection through neoadjuvant therapy [29] Regarding distant metastasis, after reviewing 838 patients with EC between 1982 and 1993, Quint indicated metastases were commonly diagnosed in the lymph nodes, liver, lung, bone, adrenal, etc., determining further management and predicting prognosis [30]. Finally, the different anatomical locations of ECs are also usually correlated to the therapeutic response and survival outcome. The upper third was commonly associated with the poor prognosis, but tended to be more sensitive to the chemoradiotherapy [21].

Based on the Fig. 4, CRT was shown to decrease the probability of CSD and to improve the survival in the high-risk group, however, no significant benefits were observed in the low-risk group. The above results indicated the necessity of selective administration of CRT to the elderly patients. Some related studies had been conducted to answer this question. As for chemotherapy, the OE0-2 trial conducted by the Medical Research Council of the United Kingdom showed that receiving regimens of cisplatin and 5-fluorouracil before surgery (n = 86) could prolong the survival of patients aged > 75 years (HR = 0.7), compared to those receiving surgery alone (n = 79) [31]. A randomized trial in Germany indicated that, compared to the double combinations of 5-fluorouracil, leucovorin, and oxaliplatin, a triple combination of 5-fluorouracil, leucovorin, oxaliplatin, and docetaxel could improve the therapeutic response and progression-free survival in patients aged 65–70 years, which was associated with a higher incidence of side effects of diarrhea (*P* = 0.006), alopecia (*P* < 0.001), neutropenia (*P* < 0.001), nausea (*P* = 0.029), and leukopenia (*P* < 0.001) [32]. Regarding radiotherapy, the CROSS trial found that chemoradiotherapy after surgery was more favorable for patients with a median age of 60 years, compared to surgery alone [33]. The median OS was 48.6 months versus 24.0 months, respectively (HR: 0.68, *P* = 0.003). Contrary to the above findings, a multicenter randomized phase III trial of FFCD 9901 involving 195 patients with a median age of approximately 60 years, investigated the effect of neoadjuvant chemoradiotherapy on patients with early stage EC and found no significant positive impact on the rate of R0 resection or survival prognosis; although the mortality after surgery was increased [34].

Generally, it is widely accepted that both the chemotherapy and radiotherapy could exert a positive effect on the survival of elderly patients with EC. For instance, after reviewing 21,593 EC patients aged ≥ 70 years from the National Cancer Database, Gregory et al. pointed out that any cancer-related treatment could play a positive role in prolonging the survival of elderly patients with EC [35]. Similarly, Daniela et al. used the SEER database (2001–2009) to show that an improved 5-year survival could be observed in elderly patients (≥ 65 years) receiving any medical or surgical therapy [36]. However, the possible limitations in these studies were that the authors did not use a competitive risk model to avoid the interference of non-CSDs in the survival analysis. Furthermore, considering the balance between benefits and harms from therapies in the elderly population, it was vital to identify the specific population that tended to benefit or suffer from the anti-tumor treatment, which was usually absent in most current studies.

The present study has several limitations. First, it was a retrospective study based on the SEER database, covering only 30% of the population of the United States [37] and with poor representation of the variable incidence and prognosis of EC worldwide [38]. Second, the qualified patients involved in our study were not sufficient to develop a strong nomogram mode with considerable net benefit and c-index; in particular, numerous cases were lost during the process of nearest neighbor matching. Third, some important information such as the quality of life, complications, and treatment protocols were absent in the SEER database; therefore, further analysis of certain specific subgroups could not be performed. Moreover, surgical margin and scope closely correlated with the prognosis of EC patients were also absent, which weakened the predictive strength and stability of the model [39]. Last, due to the lack of specific time-points for treatment, a clear causal relationship between treatment and prognosis could not be demonstrated.

## Conclusion

Based on the post-PSM cohorts, we found that CRT could decrease the probability of CSD and improve OS in elderly (≥ 70 years) EC patients in general. Further analysis of the sub-cohort marked as the high- and low-risk groups derived from a nomogram indicated that high-risk patients with tumors characterized as middle- and upper-third of the tumor location, poor and undifferentiated histological grade, ≥ 33 mm tumor size, advanced T stage, positive lymph node, and distant metastasis could be considered beneficial for aggressive anti-tumor treatment. However, for low-risk patients whose tumors were characterized as lower-third of the tumor location, well and moderately differentiated histological grade, < 33 mm of tumor size, early T stage, negative lymph node, and non-metastasis, CRT could not bring therapeutic benefits on the survival outcomes. Despite this, well-designed trials are still needed to validate our conclusions based on real-world clinical practice.

## Supplementary Information

**Additional file 1: Figure S1.** Standardized mean differences of variables between the pre- and post-matching cohorts based on the CRT or not. ^a^CRT: chemo- and radio-therapy.

**Additional file 2: Figure S2.** A, C, E: The calibration curves of nomogram for predicting 1-, 3-, and 5-year probabilities of cancer-specific death (CSD) in the training set. B, D, F: The calibration curves of nomogram for predicting 1-, 3-, and 5-year probabilities of CSD in the validation set. Nomogram-predicted CSD is plotted on the x-axis; actual CSD is plotted on the y-axis. The imaginary line indicates a perfect calibration model in which the predicted probabilities are identical to the actual incidence.

**Additional file 3: Figure S3.** Decision curve analysis (DCA) of the nomogram and 6th edition of the American Joint Committee on Cancer’s (AJCC) tumor, node, metastasis (TNM) staging system for 1-year (A), 3-year (B), and 5-year (C) overall survival. The x-axis measures the threshold probabilities, and the y-axis represents the net benefit. The horizontal line along the x-axis assumes that overall death occurred in no patients, while the solid gray line assumes that all patients will have overall death at a specific threshold probability. The blue solid line represents the 6th edition of the AJCC TNM staging system. The red solid line represents the nomogram.

## Data Availability

The data that support the findings of this study are available from the corresponding author upon reasonable request.

## Abbreviations

EC: Esophageal cancer
CRT: Chemo- and radio-therapy
SEER: Surveillance, epidemiology, and end results (database)
sHR: Sub-distribution hazard ratio
HR: Hazard ratio
CSD: Cancer-specific death
OS: Overall survival
PSM: Propensity score matching

## References

[1] Siegel RL, Miller KD, Jemal A (2020). Cancer statistics, 2020. CA Cancer J Clin.

[2] Pennathur A, Gibson MK, Jobe BA, Luketich JD (2013). Oesophageal carcinoma. Lancet (London, England).

[3] Ferlay J, Soerjomataram I, Dikshit R, Eser S, Mathers C, Rebelo M (2015). Cancer incidence and mortality worldwide: sources, methods and major patterns in GLOBOCAN 2012. Int J Cancer.

[4] Gupta B, Kumar N (2017). Worldwide incidence, mortality and time trends for cancer of the oesophagus. Eur J Cancer Prev Off J Eur Cancer Prev Organ (ECP).

[5] Huang J, Koulaouzidis A, Marlicz W, Lok V, Chu C, Ngai CH (2021). Global burden, risk factors, and trends of esophageal cancer: an analysis of cancer registries from 48 countries. Cancers (Basel).

[6] El-Serag HB, Mason AC, Petersen N, Key CR (2002). Epidemiological differences between adenocarcinoma of the oesophagus and adenocarcinoma of the gastric cardia in the USA. Gut.

[7] Muto M, Minashi K, Nihei K, Mizusawa J, Yano T, Ezoe Y (2016). Efficacy of combined endoscopic resection and chemoradiotherapy for clinical stage I esophageal squamous cell carcinoma (ESCC): a single-arm confirmatory study (JCOG0508). J Clin Oncol.

[8] Ando N, Kato H, Igaki H, Shinoda M, Ozawa S, Shimizu H (2012). A randomized trial comparing postoperative adjuvant chemotherapy with cisplatin and 5-fluorouracil versus preoperative chemotherapy for localized advanced squamous cell carcinoma of the thoracic esophagus (JCOG9907). Ann Surg Oncol.

[9] Cunningham D, Allum WH, Stenning SP, Thompson JN, Van de Velde CJ, Nicolson M (2006). Perioperative chemotherapy versus surgery alone for resectable gastroesophageal cancer. N Engl J Med.

[10] Kojima T, Muro K, Francois E, Hsu C-H, Moriwaki T, Kim S-B (2019). Pembrolizumab versus chemotherapy as second-line therapy for advanced esophageal cancer: phase III KEYNOTE-181 study. J Clin Oncol.

[11] Ajani JA, D'Amico TA, Bentrem DJ, Chao J, Corvera C, Das P (2019). Esophageal and esophagogastric junction cancers, version 2.2019, NCCN clinical practice guidelines in oncology. J Natl Compr Cancer Netw JNCCN.

[12] Zarean E, Mahmoudi M, Azimi T, Amini P (2018). Determining overall survival and risk factors in esophageal cancer using censored quantile regression. Asian Pac J Cancer Prev APJCP.

[13] Lu PP, Zhang N, Ma HM, Gu JH, Xu CL, Meng FS (2019). Study on the related factors of esophageal cancer and precancerous lesions in rural residents aged 40–69 years in Shandong Province. Zhonghua yu fang yi xue za zhi [Chin J Prev Med].

[14] Asombang AW, Chishinga N, Nkhoma A, Chipaila J, Nsokolo B, Manda-Mapalo M (2019). Systematic review and meta-analysis of esophageal cancer in Africa: epidemiology, risk factors, management and outcomes. World J Gastroenterol.

[15] Servagi-Vernat S, Créhange G, Bonnetain F, Mertens C, Brain E, Bosset JF (2017). Chemoradiation in elderly esophageal cancer patients: rationale and design of a phase I/II multicenter study (OSAGE). BMC Cancer.

[16] Won E (2017). Issues in the management of esophageal cancer and geriatric patients. Chin Clin Oncol.

[17] The Surveillance, Epidemiology, and End Results (SEER) Program. https://seer.cancer.gov/seerstat/. Accessed 27 August 2020.

[18] Gray RJ (1988). A class of K-sample tests for comparing the cumulative incidence of a competing risk. Ann Stat.

[19] Lim HJ, Zhang X, Dyck R, Osgood N (2010). Methods of competing risks analysis of end-stage renal disease and mortality among people with diabetes. BMC Med Res Methodol.

[20] Zhang Z, Kim HJ, Lonjon G, Zhu Y (2019). Balance diagnostics after propensity score matching. Ann Transl Med.

[21] Papp A, Cseke L, Farkas R, Pavlovics G, Horvath G, Varga G (2010). Chemo-radiotherapy in locally advanced squamous cell oesophageal cancer—Are upper third tumours more responsive?. Pathol Oncol Res POR.

[22] Bollschweiler E, Plum P, Mönig SP, Hölscher AH (2017). Current and future treatment options for esophageal cancer in the elderly. Expert Opin Pharmacother.

[23] Fan J, Liu Z, Mao X, Tong X, Zhang T, Suo C (2020). Global trends in the incidence and mortality of esophageal cancer from 1990 to 2017. Cancer Med.

[24] Collaborators GOC (2020). The global, regional, and national burden of oesophageal cancer and its attributable risk factors in 195 countries and territories, 1990–2017: a systematic analysis for the Global Burden of Disease Study 2017. Lancet Gastroenterol Hepatol.

[25] Arnold M, Laversanne M, Brown LM, Devesa SS, Bray F (2017). Predicting the future burden of esophageal cancer by histological subtype: international trends in incidence up to 2030. Am J Gastroenterol.

[26] Kamel MK, Lee B, Rahouma M, Harrison S, Nguyen AB, Port JL (2018). T1N0 oesophageal cancer: patterns of care and outcomes over 25 years. Eur J Cardio-Thorac Surg Off J Eur Assoc Cardio-Thorac Surg.

[27] Sakai M, Sohda M, Miyazaki T, Yoshida T, Kumakura Y, Honjo H (2018). Association of preoperative nutritional status with prognosis in patients with esophageal cancer undergoing salvage esophagectomy. Anticancer Res.

[28] Zhang H, Shang X, Ren P, Gong L, Ahmed A, Ma Z (2019). The predictive value of a preoperative systemic immune-inflammation index and prognostic nutritional index in patients with esophageal squamous cell carcinoma. J Cell Physiol.

[29] Kidane B, Korst RJ, Weksler B, Farrell A, Darling GE, Martin LW (2019). Neoadjuvant therapy vs upfront surgery for clinical T2N0 esophageal cancer: a systematic review. Ann Thorac Surg.

[30] Lagergren J, Smyth E, Cunningham D, Lagergren P (2017). Oesophageal cancer. Lancet (London, England).

[31] MRCOCW Group (2002). Surgical resection with or without preoperative chemotherapy in oesophageal cancer: a randomised controlled trial. Lancet (London, England).

[32] Al-Batran SE, Pauligk C, Homann N, Hartmann JT, Moehler M, Probst S (2013). The feasibility of triple-drug chemotherapy combination in older adult patients with oesophagogastric cancer: a randomised trial of the Arbeitsgemeinschaft Internistische Onkologie (FLOT65+). Eur J Cancer (Oxford, England: 1990).

[33] Shapiro J, van Lanschot JJB, Hulshof M, van Hagen P, van Berge Henegouwen MI, Wijnhoven BPL (2015). Neoadjuvant chemoradiotherapy plus surgery versus surgery alone for oesophageal or junctional cancer (CROSS): long-term results of a randomised controlled trial. Lancet Oncol.

[34] Mariette C, Dahan L, Mornex F, Maillard E, Thomas PA, Meunier B (2014). Surgery alone versus chemoradiotherapy followed by surgery for stage I and II esophageal cancer: final analysis of randomized controlled phase III trial FFCD 9901. J Clin Oncol Off J Am Soc Clin Oncol.

[35] Vlacich G, Samson PP, Perkins SM, Roach MC, Parikh PJ, Bradley JD (2017). Treatment utilization and outcomes in elderly patients with locally advanced esophageal carcinoma: a review of the National Cancer Database. Cancer Med.

[36] Molena D, Stem M, Blackford AL, Lidor AO (2017). Esophageal cancer treatment is underutilized among elderly patients in the USA. J Gastrointest Surg Off J Soc Surg Aliment Tract.

[37] Hayat MJ, Howlader N, Reichman ME, Edwards BK (2007). Cancer statistics, trends, and multiple primary cancer analyses from the surveillance, epidemiology, and end results (SEER) program. Oncologist.

[38] Malhotra GK, Yanala U, Ravipati A, Follet M, Vijayakumar M, Are C (2017). Global trends in esophageal cancer. J Surg Oncol.

[39] Schlick CJR, Khorfan R, Odell DD, Merkow RP, Bentrem DJ (2020). Margin positivity in resectable esophageal cancer: Are there modifiable risk factors?. Ann Surg Oncol.

