# Supplementary figures and images for: Impact of chemotherapy and radiotherapy on the survival of elderly esophageal cancer patients undergoing surgery: a SEER database analysis
Source: BMC Gastroenterol. 2021 Nov 18;21:430. doi: 10.1186/s12876-021-02016-9 (PMC8603580; doi:10.1186/s12876-021-02016-9)

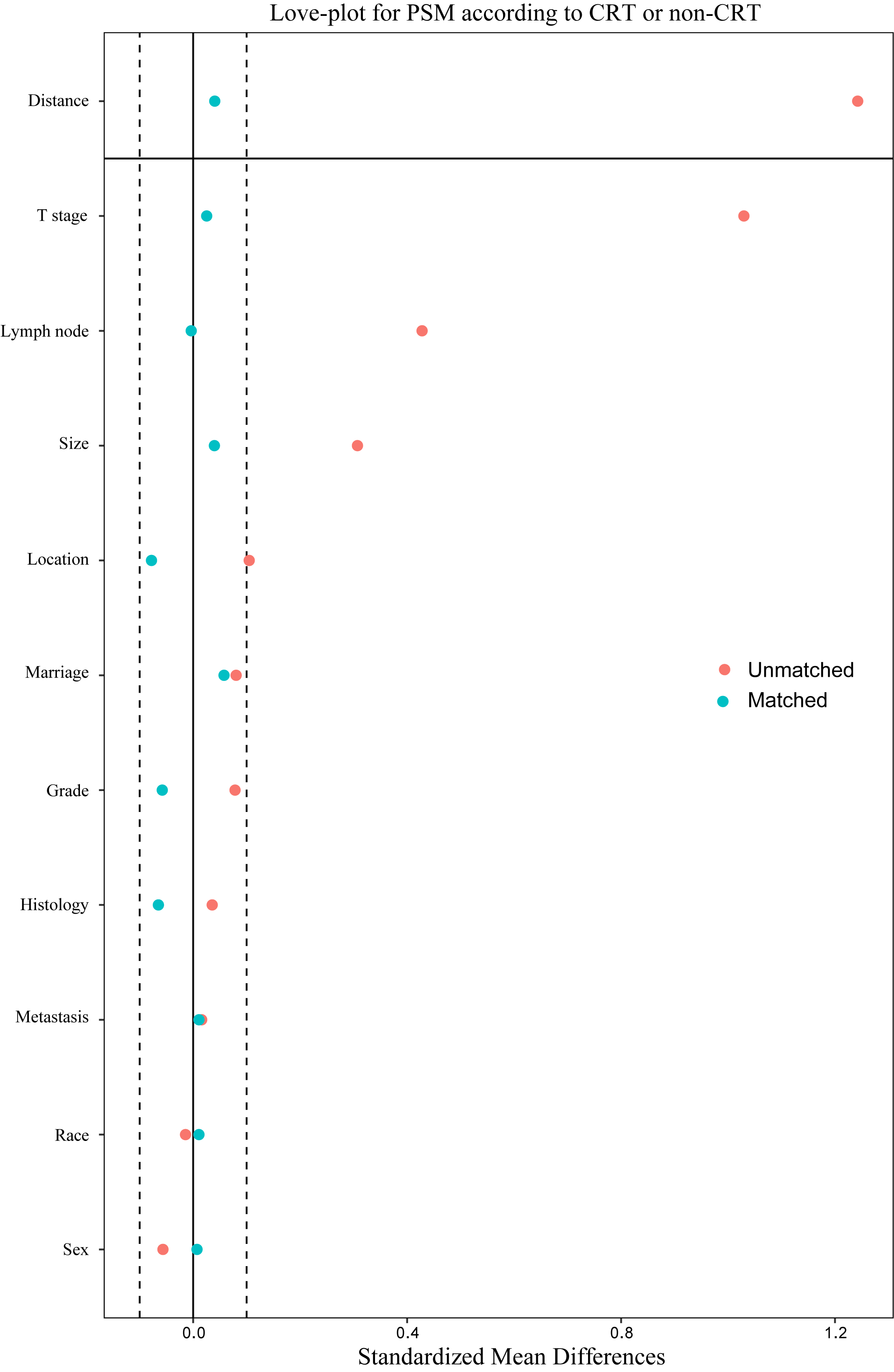

Supplement: Supplementary file 1 — Additional file 1: Figure S1. Standardized mean differences of variables between the pre- and post-matching cohorts based on the CRT or not. aCRT: chemo- and radio-therapy. [file 12876_2021_2016_MOESM1_ESM.tif]

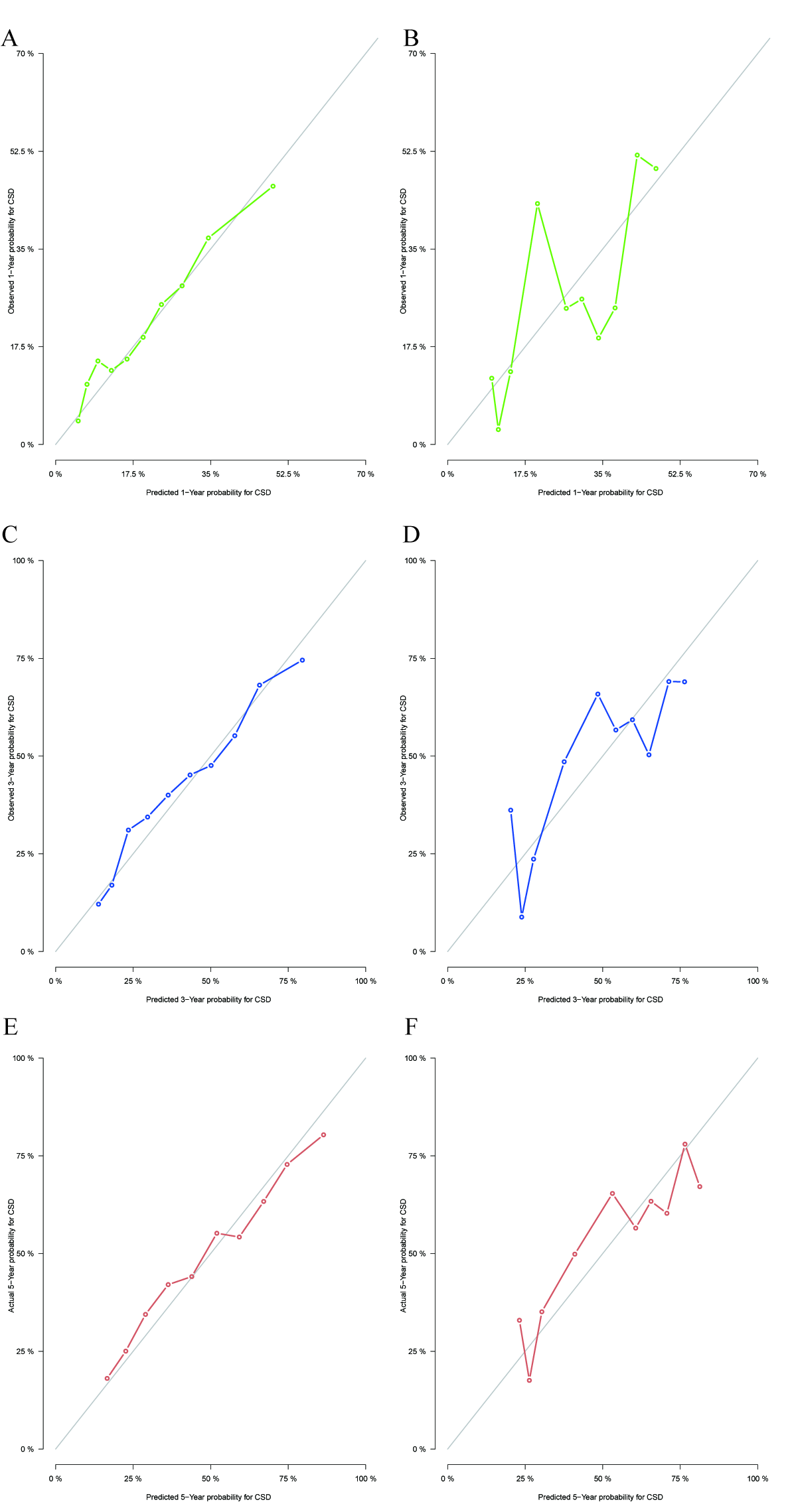

Supplement: Supplementary file 2 — Additional file 2: Figure S2. A, C, E: The calibration curves of nomogram for predicting 1-, 3-, and 5-year probabilities of cancer-specific death (CSD) in the training set. B, D, F: The calibration curves of nomogram for predicting 1-, 3-, and 5-year probabilities of CSD in the validation set. Nomogram-predicted CSD is plotted on the x-axis; actual CSD is plotted on the y-axis. The imaginary line indicates a perfect calibration model in which the predicted probabilities are identical to the actual incidence. [file 12876_2021_2016_MOESM2_ESM.tif]

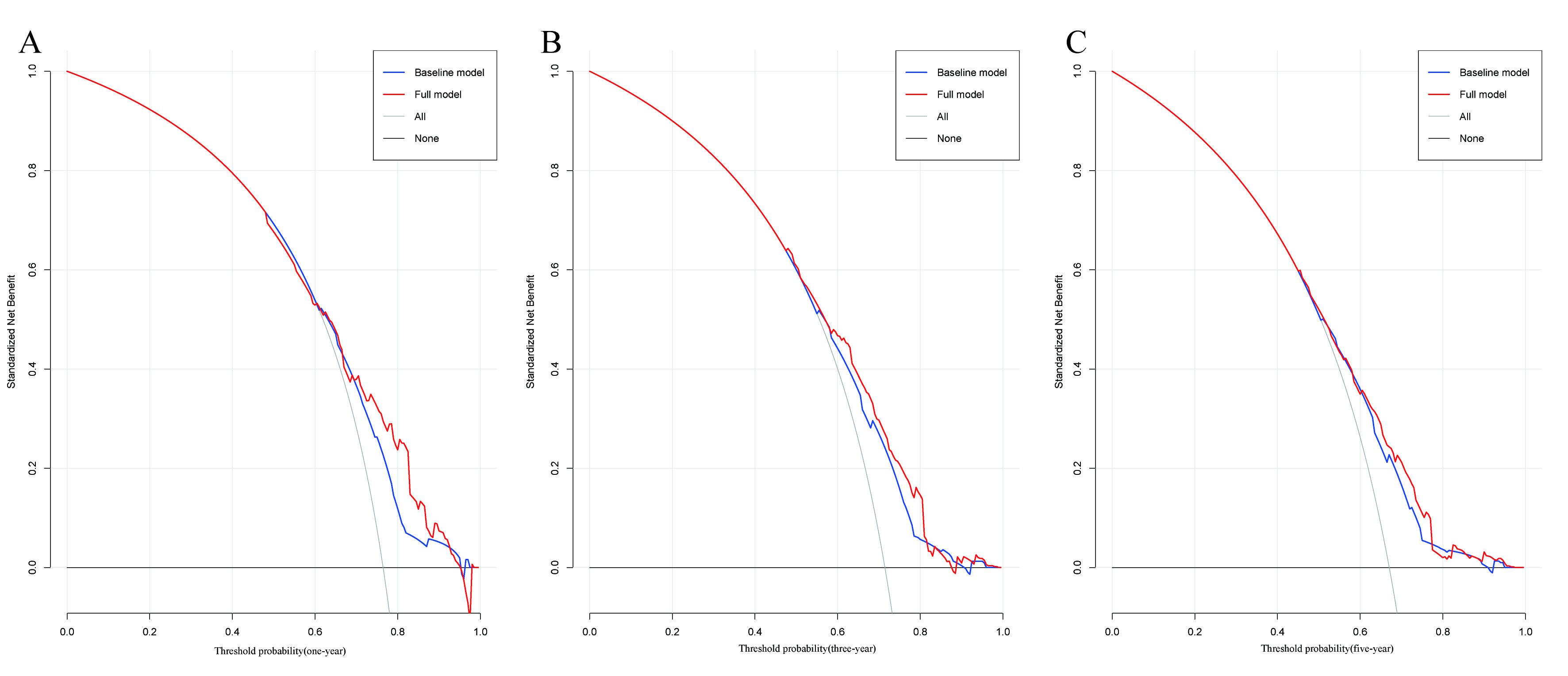

Supplement: Supplementary file 3 — Additional file 3: Figure S3. Decision curve analysis (DCA) of the nomogram and 6th edition of the American Joint Committee on Cancer’s (AJCC) tumor, node, metastasis (TNM) staging system for 1-year (A), 3-year (B), and 5-year (C) overall survival. The x-axis measures the threshold probabilities, and the y-axis represents the net benefit. The horizontal line along the x-axis assumes that overall death occurred in no patients, while the solid gray line assumes that all patients will have overall death at a specific threshold probability. The blue solid line represents the 6th edition of the AJCC TNM staging system. The red solid line represents the nomogram. [file 12876_2021_2016_MOESM3_ESM.tif]
